# Chlorogenic Acid (CGA) Isomers Alleviate Interleukin 8 (IL-8) Production in Caco-2 Cells by Decreasing Phosphorylation of p38 and Increasing Cell Integrity

**DOI:** 10.3390/ijms19123873

**Published:** 2018-12-04

**Authors:** Ningjian Liang, David D. Kitts

**Affiliations:** Food, Nutrition, and Health Program, Faculty of Land and Food Systems, The University of British Columbia, 2205 East Mall, Vancouver, BC V6T 1Z4, Canada; ningjian@mail.ubc.ca

**Keywords:** chlorogenic acid isomers, inflammation, NFκB pathway, MAPK cascades, Caco-2 cells

## Abstract

The objective of this study was to determine the effect of six chlorogenic acid (CGA) isomers known to be present in coffee and other plant foods on modulating the inflammatory response induced by pro-inflammatory cytokines in the Caco-2 human intestinal epithelial cell line. Compared to caffeoylquinic acids (CQA), dicaffeoylquinic acids (DiCQA) had significantly stronger (*p* < 0.05) capacities to reduce phosphorylation of one of mitogen-activated protein kinases (MAPK) cascades, namely p38. Compared to the control, CQA isomers treatment resulted in around 50% reduction in an interleukin-8 (IL-8) secretion, whereas DiCQA, at the same concentration, resulted in a 90% reduction in IL-8 secretion, compared to the control cells. CGA isomer treatment also showed a significant effect (*p* < 0.05) on the up-regulation of NFκB subunit p65 nuclear translocation by more than 1.5 times, compared to the control. We concluded that CGA isomers exert anti-inflammatory activity in a mixture of interferon gamma (IFNγ) and phorbol myristate acetate (PMA)-challenged Caco-2 cells, by decreasing the phosphorylation of p38 cascade and up-regulating NFκB signaling.

## 1. Introduction

Inflammatory bowel diseases (IBDs), such as Crohn’s disease and ulcerative colitis, represent chronic disorders of the gastrointestinal tract that are characterized by an abnormal immune response to antigens which lead to a persistent inflammatory state [1,2]. The cytokine responses are key pathophysiological elements that govern the initiation, evolution, and ultimately, the resolution of IBDs [3]. Cytokines are small proteins secreted mainly by immune cells that facilitate communication between cells. IBD patients have a characteristically low level of anti-inflammatory cytokines, such as interleukin-10, and an increase in pro-inflammatory cytokines, such as tumor necrosis factor alpha (TNF-α), interferon gamma (IFNγ), interleukin-6 (IL-6), interleukin-8 (IL-8), and interleukin-12 [1]. The human intestinal Caco-2 cell line has been widely used over the last twenty years to model the intestinal barrier. Under culture conditions, cells undergo spontaneous differentiation that lead to the formation of a monolayer of cells which express several morphological and functional characteristics of the mature enterocyte [4]. Our laboratory used a mixture of interferon gamma (IFNγ) and phorbol myristate acetate (PMA) to stimulate an inflammatory state in the differentiated Caco-2 cell line for the purposes of using it as an in vitro model of intestinal inflammation [5].

The transcription factor, NFκB (nuclear factor kappa-light-chain-enhancer of activated B cells) is a key regulator of inflammation [6,7]. NFκB is composed of structurally related proteins that consist of p50, p52, p65, c-RelA, and RelB, in what is known as the Rel family. Activation of NFκB is mediated through the phosphorylation of its inhibitory subunit IκB kinase complex [8]. Upon phosphorylation, IκB is degraded, releasing the NFκB subunit p65, which in turn is translocated to the nucleus and binds to specific gene promoters that modulate anti- and pro-inflammatory proteins. Gene knockout studies have shown that the NFκB pathway can have both pro- and anti-inflammatory roles. Some studies suggest that NFκB has an anti-inflammatory role by directly inhibiting the expression of genes that encode pro-inflammatory cytokines. For example, an increased p50 subunit of NFκB expression was shown to suppress TNFα production. In contrast, some studies report that NFκB have a pro-inflammatory role by enabling prolonged macrophage activation [9]. Taken together, it is possible that NFκB activation has both anti-inflammatory and pro-inflammatory roles in regulating inflammation responses depending on the physiological context.

Regulation of NFκB activity is connected with the activation of upstream protein kinases, such as mitogen-activated protein kinases (MAPKs). MAPKs are a group of serine/threonine kinases which regulate inflammation, cell survival, differentiation, and apoptosis [10]. To date, at least three MAPK cascades have been identified, including extracellular signal-regulated kinase 1 and 2 (ERK1/2), c-Jun N-terminal kinases (JNK), and p38 isomers (p38) [10]. MAPK signaling can be activated through phosphorylation of particular amino acid sequences in the protein of MAPK components, including ERK1/2, JNK, and p38. The phosphorylated MAPKs interact with other downstream components, including NFκB, which is mediated through the phosphorylation of its inhibitory subunit IκB, and subsequent degradation of IκB at the proteasome.

**Figure 1:**
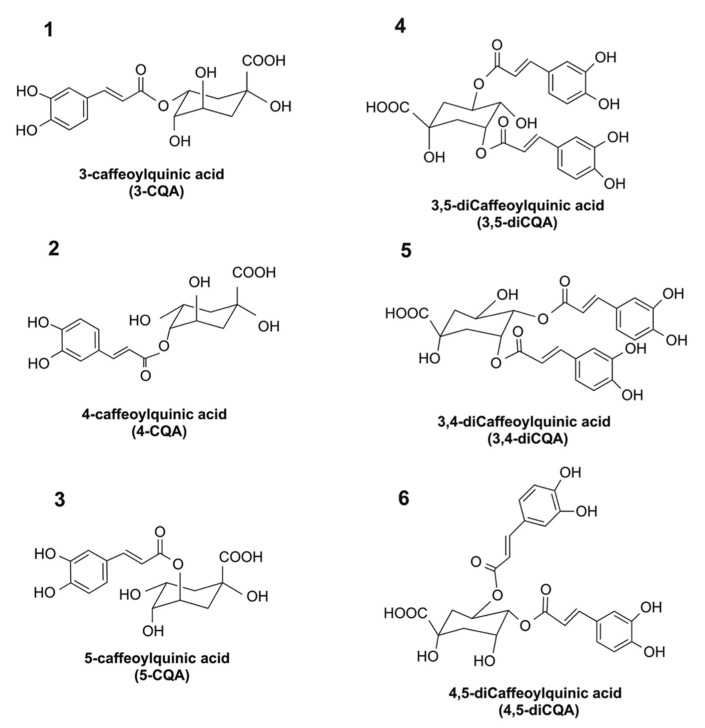
Chemical structures of chlorogenic acid (CGA) isomers (1, 2, 3, 4, 5, and 6 represent 3-caffeoylquinic acid, 4-caffeoylquinic acid, 5-caffeoylquinic acid, 3,5-dicaffeoylquinic acid, 3,4-dicaffeoylquinic acid, and 4,5-dicaffeoylquinic acid, respectively).

There is an increased interest in identifying food-derived agents that exhibit anti-inflammatory activity and which can protect the human intestine from chronic inflammation. Chlorogenic acid (CGA) is a group of phenolic acids with vicinal hydroxyl groups located on aromatic residues that are derived from esterification of cinnamic acids, including caffeic, ferulic, and *p*-coumaric acids with quinic acid. The major CGAs in coffee include mono-caffeoylquinic acids [(e.g., 3-caffeoylquinic acid (#1 in Figure 1), 4-caffeoylquinic acid (#2 in Figure 1), and 5-caffeoylquinic acid (#3 in Figure 1)] and di-caffeoylquinic acids [e.g., 3,4-dicaffeoylquinic acid (#5 in Figure 1), 3,5-dicaffeoylquinic acid (#4 in Figure 1), and 4,5-dicaffeoylquinic acid (#6 in Figure 1)]. Among these CGA isomers, 5-caffeoylquinic acid is the most abundant CGA isomer present in plants, and possesses in vitro anti-inflammatory activity [11,12,13,14,15]. Despite the fact that the bioactivity of 5-caffeoylquinic acid has been thoroughly studied, current knowledge on the anti-inflammatory activities of other CGA isomers that are present in common foods or beverages is limited.

The objectives of the current study were to examine the potential anti-inflammatory activity of different CGA isomers and the underlying molecular signaling mechanisms that are linked to anti-inflammatory activity. Specifically, experiments were conducted to compare the relative affinity of CGA isomers to influence the secretion of pro-inflammatory cytokine in the PMA + INFγ-induced Caco-2 cells model. The experimental hypothesis tested was that CGA isomers have an isomer-specific efficacy to ameliorate inflammation. In addition, the mechanisms for this response were related to isomer-specific affinity to modify NFκB signaling and phosphorylation of MAPK kinases.

## 2. Results

### 2.1. Impact of CGA Isomers on IL-8 Secretion in PMA + INFγ-Challenged Caco-2 Cells

**Figure 2:**
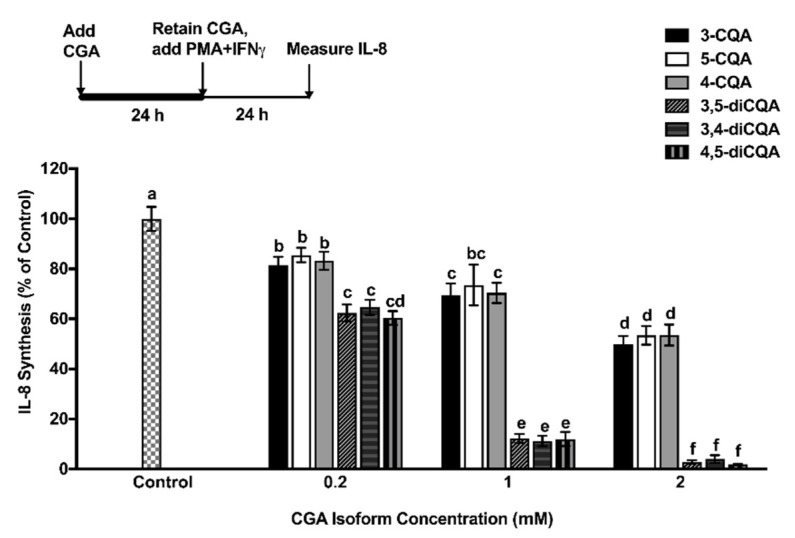
The effect of CGA isomers at different concentrations (0.2, 1, and 2 mM) on IL-8 secretion in interferon gamma (IFNγ) + phorbol myristate acetate (PMA)-challenged Caco-2 cells at a 24 h time point. Experiments were performed in triplicate, and results were expressed as mean ± standard deviation. The significance of the differences between different treatments was analyzed by a one-way analysis of variance (ANOVA), using the Bonferroni post-test with Graph Pad Prism software. Superscript with different alphabets (a, b, c, d, e, and f) are significantly different (*p* < 0.05).

Cellular uptake of CGA isomers by Caco-2 cells were confirmed by Time of Flight-Secondary ion mass spectrometry in our previous study [16]. To determine the effect of CGA isomers on mitigating the inflammatory response in Caco-2 cells, experiments were designed to treat cells with different concentrations of individual CGA isomers for 24 h in the presence of the PMA + IFNγ challenge. The time duration chosen was based on the observation that maximum IL-8 secretion following the PMA + IFNγ challenge occurred at 24 h [5]. All six CGA isomers significantly (*p* < 0.05) reduced the secretion of IL-8 level in PMA + IFNγ-challenged Caco-2 cells (Figure 2). Increasing the concentration of CQA isomer #1–3 from 0.2 mM to 2 mM resulted in a 18% and 50% reduction in IL-8 secretion, respectively, compared to the control. Repeating the experiment with diCQA isomers #4, 5, and 6 at 2 mM produced more than 90% inhibition of IL-8 secretion, compared to the controls.

### 2.2. Transepithelial Electrical Resistance (TEER) Values in Caco-2 Monolayers

**Figure 3:**
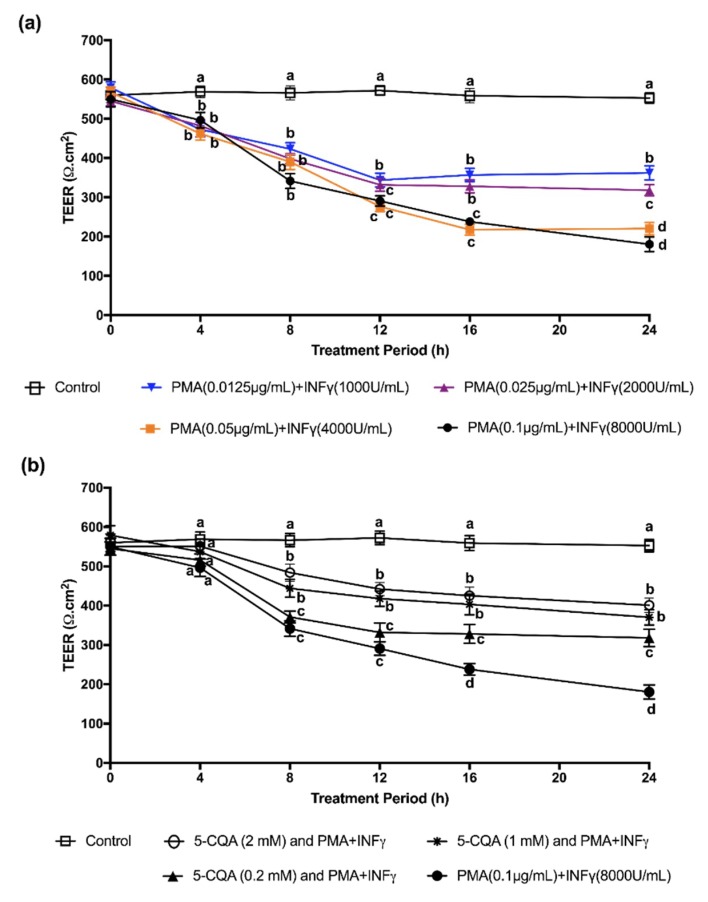
(**a**) TEER values of Caco-2 challenged by various concentrations of PMA + IFNγ for various periods. Significance of differences between values at same time point analyzed by ANOVA using the Bonferroni post-test. Superscript with different alphabets (a, b, c, and d) are significantly different (*p* < 0.05) at the same treatment time among different treatments. (**b**) Effects of 5-CQA on paracellular permeability of Caco-2 cell monolayers induced by PMA (0.1 μg/mL) + IFNγ (8000 U/mL) and incubated with or without 0.2, 1, or 2 mM 5-CQA for various periods. TEER values are shown as means ± SD. Significance of differences between values at same time point analyzed by ANOVA using the Bonferroni post-test. Superscripts with different alphabets (a, b, c, and d) are significantly different (*p* < 0.05).

We assessed the effects of various concentrations of PMA + IFNγ on Caco-2 cells as a functional test of changes in monolayer integrity. We found that PMA + IFNγ altered the integrity of Caco-2 cell monolayers, as shown by a decreased TEER value. Figure 3a shows that the TEER value declined from 550 ± 22 to 341 ± 26 Ω·cm^2^ after 8 h of incubation with PMA (0.1 μg/mL) + IFNγ (8000 U/mL), and then declined further to 181 ± 18 Ω·cm^2^ after 24 h in both apical and basolateral compartments. This combination of PMA and IFNγ produced a maximal (*p* < 0.05) response in TEER value decline compared to the control, and was therefore chosen as the optimal condition to test the effect of CGA on changes in TEER values.

Equimolar concentrations of different CGA isomers produced similar capacities to attenuate the reduction in TEER values of Caco-2 cells when induced with PMA + IFNγ. Therefore, we will only be presenting the 5-caffeoylquinic acid (5-CQA) data (Figure 3b). For example, a concentration of 0.2 mM, 5-CQA resulted in a TEER value of Caco-2 cell monolayers that was 37% higher than monolayers incubated with only PMA + IFNγ only after 16 h incubation. Increasing the concentration of 5-CQA to 1 mM improved TEER values at 8 h. These findings demonstrate that the reduced inflammatory changes in inflamed Caco-2 cells attributed to the CGAs corresponded to improved function of intestinal integrity, as assessed by TEER values.

### 2.3. CGA Isomers Up-Regulated the NFκB Signaling Pathway in PMA + INFγ-Challenged Caco-2 Cells

**Figure 4:**
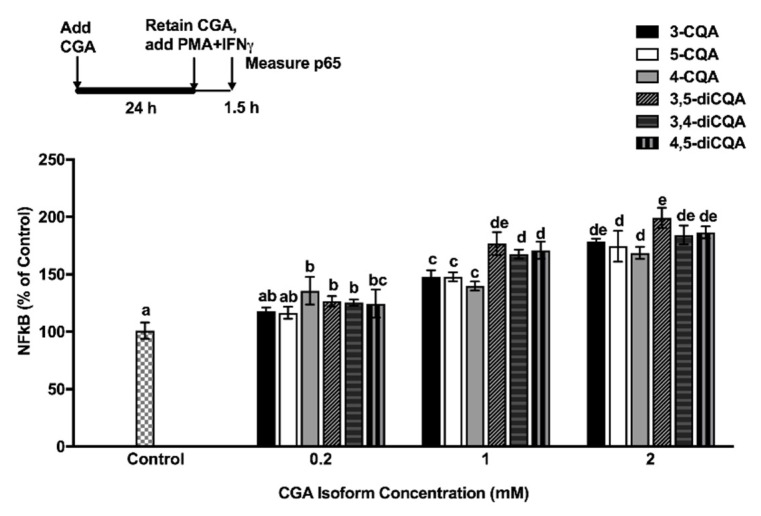
Effect of CGA isomers on p65 nucleus translocation (% of control) in PMA + INFγ-induced Caco-2 cells. Experiments were performed in triplicate, and results were expressed as mean ± standard deviation. The significance of the differences between different treatment was analyzed by ANOVA, using a Bonferroni post-test with Graph Pad Prism software. Value of *p* < 0.05 was considered to be statistically significant. Superscript with different alphabets (a, b, c, d, and e) are significantly different (*p* < 0.05).

The effect of CGA isomers on the NFκB signaling pathway in Caco-2 cells was examined at 1.5 h after a PMA + INFγ challenge. In a previous study, peak activation of NFκB following treatment of Caco-2 cells with a PMA + INFγ challenge occurred at 1.5 h [17]. Other previous studies reported that phenolic compounds had the potential to exert anti-inflammatory activity by down-regulating NFκB signaling [13,14,15]. However, data presented herein shows that CGA isomers (at 1 mM and 2 mM) were effective at significantly up-regulating NFκB subunit p65 nuclear translocation by more than 1.5 times the control values (Figure 4).

### 2.4. Temporal Effects of the PMA + IFNγ Challenge in MAPK Signaling in Caco-2 Cell

**Figure 5:**
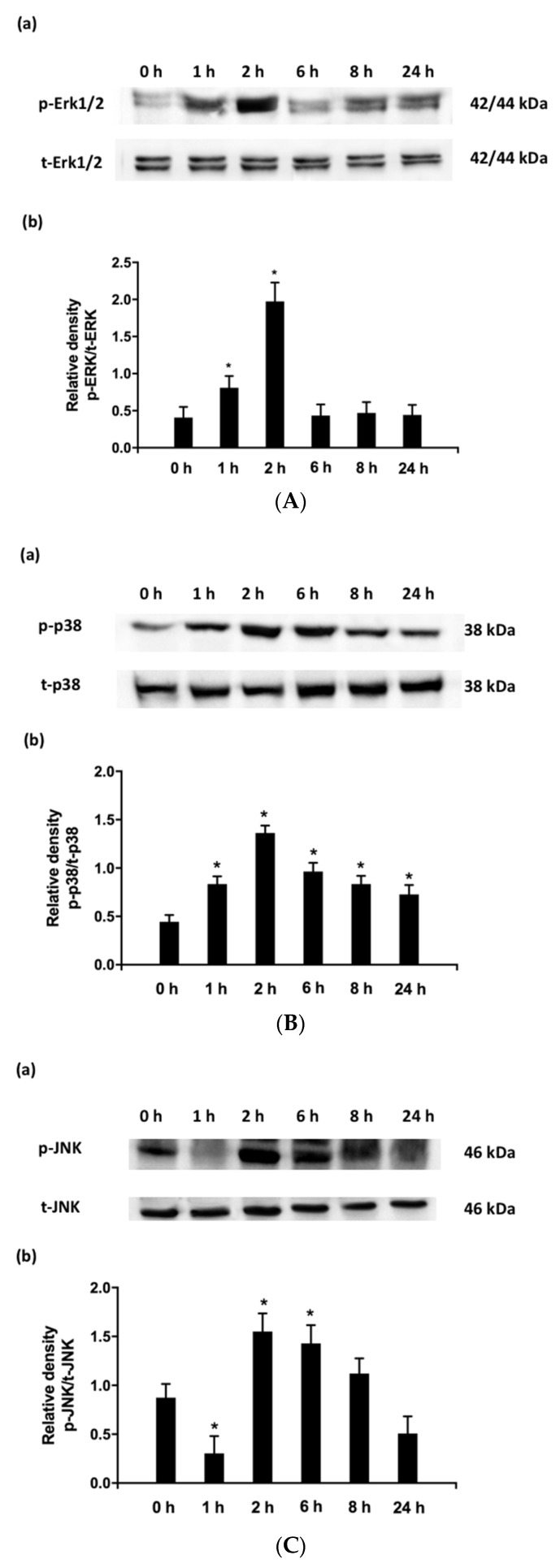
(**A**) (a) Western Blotting analysis showing the time-dependent effects of PMA + IFNγ (inducer) on the expression of ERK1/2 (p = phosphorylated, t = total, ERK1 is 42 kDa, ERK2 is 44 kDa) in Caco-2. Please see the picture of full gel in Supplementary Figures S1 and S2. (b) Densitometry analysis determined from western blots. Phosphorylated (p-ERK1/2) relative to total (t-ERK1/2). Values represent mean ± SD (*n* = 3). * represents significant difference compared to 0 h by student’s *t* test, *p* < 0.05. (**B**) (a) Western Blotting analysis showing the time-dependent effects of PMA + IFNγ (inducer) on the expression of p38 (p = phosphorylated, t = total) in Caco-2. Please see the picture of full gel in Supplementary Figures S3 and S4. (b) Densitometry analysis determined from western blots. Phosphorylated (p-p38) relative to total (t-p38). Values represent mean ± SD (*n* = 3). * represents significant difference compared to 0 h by student’s *t* test, *p* < 0.05. (**C**) (a) Western Blotting analysis showing the time-dependent effects of PMA + IFNγ (inducer) on the expression of JNK (p = phosphorylated, t = total) in Caco-2. Please see the picture of full gel in Supplementary Figures S5 and S6. (b) Densitometry analysis determined from western blots. Phosphorylated (p-JNK) relative to total (t-JNK). Values represent mean ± SD (*n* = 3). * represents significant difference compared to 0 h by student’s *t* test, *p* < 0.05.

Treating Caco-2 cells with pro-inflammatory cytokines resulted in changes in ERK 1/2 phosphorylation (Figure 5A); p-p38/t-p38 (Figure 5B); and p-JNK/t-JNK (Figure 5C). The phosphorylation of ERK 1/2 was first observed at 1 h following the PMA + IFNγ challenge, and reached its maximum expression after 2 h. The observed effect on p38 phosphorylation began early within the first hour and reached maximum expression at 2 h after the PMA + IFNγ challenge, before gradually decreasing 6 h thereafter. The expression of p-JNK declined slightly during the first hour of the PMA + IFNγ challenge and reached its highest level at 2 h, showing no further change up to 6 h. The expression of p-JNK declined to the level observed in normal healthy cells 8 h after the cytokine challenge.

### 2.5. Modulation of MAPK Signaling by CGA Isomers

**Figure 6:**
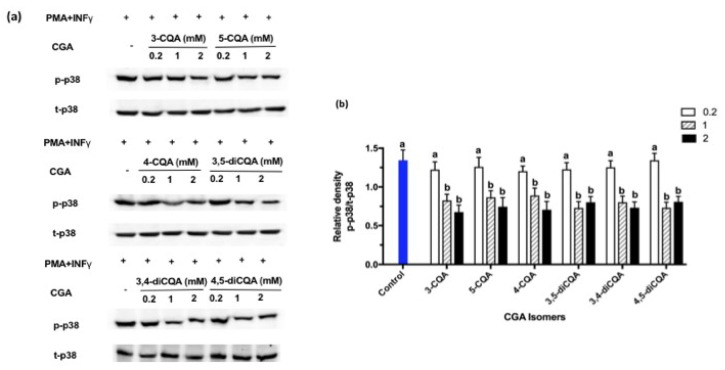
(**a**) Representative Western Blotting gel showing the effects of different CGA isomers on the expression of p38 (p = phosphorylated, t = total) in Caco-2. (**b**) Densitometry analysis determined from western blots. Phosphorylated (p-p38) relative to total (t-p38). Values represent mean ± SD (*n* = 3). The significance of the differences between different treatment was analyzed by one-way analysis of variance (ANOVA), using the Bonferroni post-test with Graph Pad Prism software. Superscript with different alphabets (a and b) are significantly different (*p* < 0.05).

The effect of individual CGA isomers on MAPK signaling was studied in PMA + IFNγ-challenged Caco-2 cells. Based on the results obtained from the time-course study, we examined the effect of CGA isomers on the phosphorylation of Erk1/2 at 2 h; p38 at 2 h, and the JNK cascade at 6 h, respectively. There were no significant changes in REK and JNK phosphorylation with CGA isomer treatment to Caco-2 cells, compared to controls. However, the CGA isomers treatment produced a concentration dependent, and isomer independent decrease in p38 phosphorylation when expressed as p-p38/t-p38, compared with the control group (*p* < 0.05) at 1–2 mM (Figure 6). No significant differences were observed for p-p38/t-p38 expression that could be attributed to the six individual CGA isomers when treated at the same concentration.

## 3. Discussion

A wide range of CGA concentrations (e.g., 0.7 mM to 30 mM) are known to exist in different coffee brews, depending on the source of beans and how it is prepared for consumption [18,19]. Taking into consideration the dilution of CGAs in luminal fluids, we chose to examine CGA at a concentration range of 0.2–2 mM, which could still exist in the intestinal lumen after coffee consumption.

The gut epithelium represents an important frontline cellular component of the innate immune system. Human epithelial cells that are challenged with inflammatory mediators have an important role in elucidating factors that contribute to inflammatory bowel diseases. For instance, interferon γ (INFγ), lipopolysaccharide (LPS), tumor necrosis factor α (TNFα), and PMA are examples of commonly used inflammatory mediators that trigger inflammation in different intestinal and non-intestinal cell models. IFNγ primarily signals through the JAK-STAT pathway, which involves sequential receptor recruitment and activation of kinases that control transcription of target genes via specific response elements [20]. LPS, which is the major component of the outer membrane of Gram-negative bacteria, bind to TLR4 and triggers the secretion of pro-inflammatory mediators [21]. TNFα also plays a central role in triggering inflammation by interacting with receptors such as TNFR1 and TNFR2 [22]. PMA is a potent activator of protein kinase C (PKC) [23]. In this study, we used a cocktail of IFNγ and PMA to trigger an inflammation response in differentiated Caco-2 cells [5]. In this model, interleukin 8 (IL-8) was significantly secreted. The amount of other pro-inflammatory cytokines was also measured, but absolute levels were not significantly different compared to the control. IL-8 is a pro-inflammatory cytokine that consists of 99 amino acids with a molecular mass of 10 kDa. Clinical studies have provided evidence to show that mucosal IL-8 protein concentration is not detectable in non-IBD patients, but is increased in subjects with IBD [24]. Others reported that the lamina propria of the colon in IBD patients secrete significantly higher levels of IL-8 compared to healthy controls [25]. Using an anti-cytokine approach that targets specific pro-inflammatory cytokines is also an effective therapy to alleviate clinical symptoms of IBD. In the current study, our goal was to explore the possible protective effects of a dietary component, such as CGA isomers, on inflamed Caco-2 cells targeted at an inflammatory biomarker, IL8.

Associations between oxidative stress and changes in tight junction proteins have been explored in detail. For example, TEER values of Caco-2 and T-84 cell monolayers are both decreased by H_2_O_2_ generated by the xanthine/xanthine oxidase system [26]. Furthermore, increased intracellular ROS induced in Caco-2 cells by the bile acid and cholic acid impairs tight junction proteins, which in turn decreases the Caco-2 cell monolayer TEER value [27]. Barrier integrity is disrupted with a 25% decline in TEER values in Caco-2 monolayers incubated in the presence of TNF-α [28]. These findings show that both ROS and inflammatory cytokines impair the functionality of the epithelial barrier, which comprises an optimal assembly of tight junction proteins [29]. This explains the declining TEER values of Caco-2 cells induced by PMA + IFNγ in our study. The ability of CGA to protect the barrier integrity of intestinal epithelial cells is similar to other studies that have tested the capacity of another polyphenolic compound, quercetin, to protect TEER values of Caco-2 cells [30,31].

CGA isomers were also shown to up-regulate the NFκB signaling pathway in PMA + INFγ- challenged Caco-2 Cells. This result demonstrates that the anti-inflammatory activity of CGAs occurred in a concentration-dependent manner for all isomers. Moreover, dicaffeoylquinic acids had significantly (*p* < 0.05) higher anti-inflammatory activity compared to caffeoylquinic acids, with concentrations of 1 mM and 2 mM, respectively. Our finding supports other studies, where the anti-inflammatory effect of CQA isomer #3 on TNF-α- and H_2_O_2_-induced Caco-2 cells was reported over a concentration range of 0.5 to 2 mM [24]. Our results also confirm and extend the knowledge that anti-inflammatory activity for different CGA isomers exists in differentiated, inflamed Caco-2 cells.

Some studies have shown that NFκB activity will increase after exposure to H_2_O_2_, which was linked to the activation of the IκB kinase complex that leads to p65 nucleus translocation [32]. To interpret the present observation, we propose that the effect of CGA isomers to up-regulate NFκB signaling is linked to an affinity to donate an electron, or electrons, to free radicals that were induced by the PMA + INFγ challenge. The CGA isomers thus become phenoxyl radicals (ArO·), which in turn act to modulate NFκB signaling. Support for this explanation comes from the fact that the antioxidant capacity of plant phenolics is attributed to an affinity to trap the chain-carrying peroxyl radicals (ROO·), forming a hydroperoxide (ROOH) and derived, resonance-stabilized phenoxyl radicals (ArO·) (Reaction (1)) [33]. The derived phenoxyl radical has the capacity to react with another peroxyl radical to form non-radical products, which terminates the reaction (Reaction (2)), or alternatively, reacts again to produce another peroxyl radical (Reaction (3)) [33]:

ROO· + ArOH → ROOH + ArO·(1)

ROO· + ArO· → nonradical products(2)

ArO· + RH → ArOH + R· → ROO·(3)

where ROO· represents peroxyl radicals; ROOH represents mono-substituted derivative of hydrogen peroxide; ArOH represents phenolic acid; ArO· represents phenoxyl radicals; RH represents an oxidation site adjacent to a double bond in any unsaturated fatty acid; and R· represents the free radical formed.

In the present study, following the uptake of CGA isomers by Caco-2 cells, the intracellular ROS induced by a PMA + IFNγ challenge was reduced. This can be attributed to an affinity of CGA isomers to donate an electron/electrons, thus quenching free radicals, such as hydroxyl (OH·), superoxide (O_2_·), nitric oxide (NO·), peroxyl radicals (ROO·), and lipid peroxyl (LOO·). These radicals individually or collectively contribute to the onset of oxidative stress. To describe the antioxidant activity of CGA isomers, it is important to recognize that phenoxyl radicals (ArO·) are formed in the reaction (Reaction 1). Employing electron paramagnetic resonance (EPR) techniques have demonstrated the existence of phenoxyl CGA radicals by showing the EPR spectra of both a primary and secondary CGA radical in a H_2_O_2_ peroxidase reaction [34]. In relative terms, the newly formed CGA phenoxyl radical is not as active as other ROS species, such as OH·, O_2_·-, NO·, ROO·, and LOO·, but nevertheless could contribute to modifying cell redox biology. Thus, CGA radicals (ArO·), if not reduced, could become a low-level pro-oxidant which promotes NFκB activation, before reacting with peroxyl radicals (ROO·) to form a non-radical product. This explanation supports our observation that an increase in NFκB occurred initially when CGAs were incubated with Caco-2 cells that had undergone induced oxidative stress.

MAPK cascades are activated by a family of dual-specificity kinases that phosphorylate MAPK cascades at specific Thr and Tyr amino acid residues. A broad range of extracellular stimuli, including cytokines, mitogens, growth factors, and environmental stressors, are known to activate the phosphorylation of MAPK cascades in differentiated Caco-2 cells. Activation of MAPKs is controlled via membrane-associated signaling complexes, and involves a network that includes Ras proteins(s), the Raf family of serine kinases, and MAPK kinases. Previous work has shown that phorbol 12-myristate 13-acetate (PMA) can activate the ERK1/2 and p38 phosphorylation in differentiated Caco-2 cells [35]. Another study reported that INFγ also activates MAPK signaling in macrophages [36]. In the present study, we demonstrated that the cocktail of PMA + IFNγ was effective at triggering the phosphorylation of ERK1/2, p38, and JNK cascades in differentiated Caco-2 cells.

Solid evidence exists that the modulation of p38 has a critical role in inflammatory responses [37]. A major function of p38 is to control the production of pro-inflammatory cytokines. In IBD patients, for example, the expression of p-p38 increases [37]. After the phosphorylation of p38, pro-inflammatory cytokines that include interleukin 1β and TNF-α increase, which leads to a stimulation in pro-inflammatory cytokine production [37]. This will result in further p38 phosphorylation and ultimately lead to an inflammatory response [38]. Therefore, controlling p38 phosphorylation is an effective strategy to control inflammation. The p38 inhibitory compounds’ efficacy at controlling the secretion of pro-inflammatory cytokines in IBD patients has been attempted. For example, Docena et al. [39] showed that p38 inhibitory drugs reduce pro-inflammatory cytokines (TNF-α, IL-1β, and IL-6) from lamina propria mononuclear cells and biopsies [39]. The results presented in Figure 5 show that CGA isomers inhibit p-p38, which leads to a suppression of cytokines that are involved in inflammation. Other signaling pathways are probably also involved in these responses.

## 4. Materials and Methods

### 4.1. Reagents

3-(4,5-dimethylthiazol-2-yl)-2,5-diphenyl tetrazolium bromide (MTT), minimum essential medium (MEM), IFNγ, PMA, sodium dodecyl sulfate (SDS), and bovine serum albumin (BSA), were purchased from Sigma (St. Louis, MO, USA). All CGA isomers, (#1 to #6) were obtained from the Cerillian Corporation (Round Rock, TX, USA) and Chengdu Must Bio-Technology Co. (Chengdu, Sichuan, China). Fetal bovine serum (FBS), penicillin, and streptomycin were purchased from Gibco^®^ (Grand Island, NY, USA). The Nuclear Extraction Kit and p65 Transcription Factor Assay Kit was purchased from the Cayman Chemical Company (Ann Arbor, MI, USA).

### 4.2. Cell Culture

The human colon adenocarcinoma cell line Caco-2 cells (HTB-37, American Type Culture Collection, Manassas, VA, USA) were cultured with minimum essential medium, containing 10% (*v*/*v*) fetal bovine serum, 100 U/mL penicillin, and 100 µg/mL of streptomycin. Cultures were maintained at 37 °C in a humidified incubator containing 5% (*v*/*v*) CO_2_, and the medium was changed every two to three days. For individual experiments, cells were seeded into 6- or 96-well plates at a density of 1 × 10^5^/cm^2^ for 21 days to allow for spontaneous differentiation.

### 4.3. In Vitro Model of Intestinal Inflammation

The cells were challenged with a mixture of PMA (0.1 µg/mL) + IFNγ (8000 U/mL) (PMA + IFNγ) for 24 h to trigger inflammation after each differentiation and serve as an in vitro model of intestinal inflammation [16]. In this model, the secretion of 10 cytokines (interleukin 1A, interleukin 1B, interleukin 2, interleukin 4, interleukin 6, interleukin 8, interleukin 10, interleukin 12, interleukin 17A, tumor necrosis factor α) were analyzed; however, only the results of interleukin 8 was found to be significant and were therefore reported in this study.

### 4.4. Experiment Treatment

For CGA isomer treatment, Caco-2 cells were cultured to differentiation and were pre-incubated with MEM containing individual CGA isomers at 0.2, 1, and 2 mM for 24 h. Then, the medium was replaced with media containing a fresh CGA and PMA + IFNγ cocktail. To ensure sterilization, CGA isomers were dissolved in MEM and sterilized by passage through a 2-µm filter before being added to Caco-2 cells. The controls were cells incubated with PMA + IFNγ, but not CGA isomers. The blanks comprised of Caco-2 cells which were either incubated without CGA isomers or PMA + IFNγ.

### 4.5. Assessment of Cell Viability

Cellular metabolic activity is an indirect measure of viability and was assessed using MTT using 96-well plates, as described previously [16].

### 4.6. Effect of CGA Isomers on IL-8 Secretion in PMA + INFγ Challenged Cell

Caco-2 cells were treated as described in Section 4.4, and the supernatant was collected at 24 h. The Human IL-8 Single Analyte ELISA kit (Qiagen, Hilden, Germany) was used to quantify the IL-8 level in the culture medium. Specific protocols were described in the manufacturer’s instruction booklet. The measurement of each media sample was performed in triplicate.

### 4.7. TEER Measurement

Caco-2 cells were cultured on semipermeable filter inserts (Falcon^®^ Permeable Support) with a 0.4 μm translucent, high-density PET Membrane (Corning Life Sciences, Tewksbury, MA, USA) designed for 24-well plates. PMA + IFNγ was added to differentiated Caco-2 cells in both the apical and basolateral compartments, respectively, and the electrical resistance between the compartments was then measured using a Millicell^®^ ERS voltammeter (Millipore Ltd., Etobicoke, ON, Canada). One electrode was placed in each of the upper apical and lower basolateral compartments, which were separated by the Caco-2 monolayer. The procedure included measuring the resistance (R_Blank_) of the semipermeable membrane without any cells (blank) and measuring the resistance across the cell layer on the semipermeable membrane (R_Total_). TEER values are reported in units of Ω·cm^2^, and were calculated as:

(R_Total_ − R_Blank_) (Ω) × M_Area_ (cm^2^) (4)

The effects of CGA isomers on the TEER values of Caco-2 cells treated with PMA + IFNγ were also evaluated.

### 4.8. Analysis of p65 Binding Activity by Transactivation Assay

To study the role of different CGA isomers on modulating NFκB signaling in IFNγ + PMA-challenged Caco-2 cells, the binding efficacy of the nuclear extract with the NFκB consensus binding site was measured using a NFκB (p65) Transcription Factor Assay Kit (10007889, Cayman Chemical Company, Ann Arbor, MI, USA). Briefly, Caco-2 cells were first recovered with a rubber spatula into an ice-cold PBS in the presence of phosphatase inhibitors after treatment with the CGA isomer, as described in the *Experiment treatment* section. The cytosolic fraction and nuclear fraction were extracted and separated using a Nuclear Extraction Kit (10009277, Cayman Chemical Company, Ann Arbor, MI, USA) according to the manufacturer’s instructions. Purity of the nuclear fraction was confirmed with the presence of histone, though only in the nuclear fraction and not in the cytoplasm fraction; GAPHD was present only in the cytoplasm and not in the nuclear fraction. Protein concentrations were determined using the BCA protein assay, and of all the samples were adjusted to 2 mg/mL. An aliquot of the cellular protein extract, 10 µL (containing 20 µg of protein), was incubated with immobilized oligonucleotides containing the NFκB consensus binding site (5’-GGGACTTTCC-3’). The active form of p65 that bound to the oligonucleotides was detected using an anti-p65 primary antibody (1:1000 dilution) for 1 h, followed by incubation with an HRP-conjugated secondary antibody (1:1000 dilution) for 1 h at room temperature. Absorbance values were recorded using a microplate reader set at 450 nm.

### 4.9. Western Blot to Analyze p-Erk 1/2, p-JNK, p-p38, t-Erk 1/2, t-JNK, and t-p38 (p = Phosphorylated; t = Total)

MAPK pathways are involved in the regulation of gene expression of several proteins involved in inflammation. To further investigate the mechanisms underlying the anti-inflammatory effects of CGA isomers, a western blot analysis was performed to show activation of MAPKs. A time-course experiment was performed in differentiated Caco-2 cells in the presence of PMA + IFNγ at times that consisted of 0, 1, 2, 6, 8, and 24 h to monitor MAPK signaling. The expression of different phosphorylated forms of MAPKs (p-ERK1/2, p-p38, and p-JNK) and total ERK1/2 (t-ERK), total p38 (t-p38), and total JNK (t-JNK) were analyzed by western blot.

M-PER™ Mammalian Protein Extraction Reagent (Cat. No 78501, Thermo Fisher Scientific, Waltham, MA, USA) (added phenyl methyl sulphonyl fluoride and protease inhibitor) was used to extract the Caco-2 cell protein, according to the manufacturer’s instructions. The total protein extracts (20 µg protein) were subjected to 12% sodium dodecyl sulfate-polyacrylamide gel electrophoresis (SDS-PAGE) using a Mini-PROTEAN^®^ II Multiscreen Apparatus (Bio-Rad Laboratories, Hercules, CA, USA). Separated proteins were transferred to nitrocellulose membranes (0.2 µm, 7 × 8.4 cm; Bio-Rad Laboratories, Hercules, CA, USA), and the membranes were incubated with 3% BSA at 20 °C for 1 h to block unspecific binding sites, before incubating with different antibodies (for detecting phosphorylated MAPK cascades): anti-phospho-Erk1/2 (phospho Thr202/Tyr204) (D13.14.4E) XP^®^ Rabbit mAb (1:2000 dilution) (4370S, Cell Signaling Technology, Danvers, MA, USA); anti-phospho-JNK1 + JNK2+ JNK3 (phospho T183 + T183 + T221) antibody (1:1000 dilution) (ab124956, Abcam Inc., Ontario, Canada); anti-phospho-p38 MAPK (phospho Thr180/Tyr182) (D3F9) XP^®^ Rabbit mAb (1:1000 dilution) (4511S, Cell Signaling Technology, Danvers, MA, USA) at a temperature of 4 °C, overnight. Subsequently, the membranes were washed three times using Tris-buffered-saline-Tween (TBST) and then incubated with a secondary antibody, a horse-radish peroxidase-conjugated goat anti-rabbit IgG antibody (1:1000 dilution) (Invitrogen, Carlsbad, CA, USA) for 1 h. The membranes were then washed three times with TBST, and reacted against a Western ECL substrate (Bio-Rad Laboratories Inc., Woodinville, WA, USA) for 3 min. A ChemiDoc™ MP Imaging System (Bio-Rad Laboratories Inc., Woodinville, WA, USA) was used to detect the proteins. Equal protein loading was demonstrated by stripping the original blot and re-probing with t-ERK1/2, t-JNK, and t-p38 antibodies. Specifically, the membrane that was used for the detection of p-Erk1/2 was washed three times with TBST and incubated in a mixture antibody solution that contained both anti-phospho-Erk1/2 (phospho Thr202/Tyr204) (D13.14.4E) XP^®^ Rabbit mAb (1:2000 dilution) (4370S, Cell Signaling Technology, Danvers, MA, USA) and anti-Erk1/2 (137F5) Rabbit mAb (1:1000 dilution) (4695S, Cell Signaling Technology, Danvers, MA, USA). Similarly, the membrane that was used for the detection of p-JNK was washed three times with TBST and incubated in a mixture antibody solution that contained both anti-phospho-JNK1 + JNK2 + JNK3 (phospho T183 + T183 + T221) antibody (1:1000 dilution) (ab124956, Abcam Inc., Ontario, Canada) and anti-JNK1 + JNK2 + JNK3 antibody (1:2000 dilution) (ab208035, Abcam Inc., Ontario, Canada). Similarly, the membrane that was used for the detection of p-p38 was washed three times with TBST and incubated in a mixture antibody solution that contained both anti-phospho-p38 MAPK (phospho Thr180/Tyr182) (D3F9) XP^®^ Rabbit mAb (1:1000 dilution) (4511S, Cell Signaling Technology, Danvers, MA, USA), and anti-p38 MAPK (D13E1) XP^®^ Rabbit mAb (1:1000 dilution) (8690S, Cell Signaling Technology, Danvers, MA, USA. Image Lab. 4.1 software (Bio-Rad Laboratories Inc., Woodinville, WA, USA) was used to conduct the densitometry analysis. The expression of p-ERK1/2 was normalized by calculating the relative density of that which was phosphorylated (p-ERK1/2) to the total (t-ERK1/2). Similarly, the relative density of the phosphorylated (p-JNK) to the total (t-JNK), and the relative density of the phosphorylated (p-p38) to the total (t-p38), were also calculated. The normalized expression was then compared across samples to detect the impact of CGA isomers on MAPK signaling.

### 4.10. Statistics

All experiments were performed in triplicate wells for each condition, and repeated at least three times. Representative data are presented as mean ± standard deviation (SD). The data were analyzed by a one-way ANOVA using Graphpad Prism software (San Diego, CA, USA). Significant differences were compared using Bonferroni post-hoc tests with *p* < 0.05 representing a statistically significant difference.

## 5. Conclusions

We concluded that CGA isomers exerted anti-inflammatory activity in IFNγ + PMA-challenged Caco-2 cells in part, by decreasing the phosphorylation of the p38 MAPK cascade and up-regulating NFκB signaling.

## Supplementary Materials

The following are available online at http://www.mdpi.com/1422-0067/19/12/3873/s1, Figure S1: Full image of Western Blotting analysis showing the time-dependent effects of PMA + IFNγ (inducer) on the expression of p-Erk1/2. Figure S2: Full image of Western Blotting analysis showing the time-dependent effects of PMA + IFNγ (inducer) on the expression of t-Erk1/2. Figure S3: Full image of Western Blotting analysis showing the time-dependent effects of PMA + IFNγ (inducer) on the expression of p-p38. Figure S4: Full image of Western Blotting analysis showing the time-dependent effects of PMA + IFNγ (inducer) on the expression of t-p38. Figure S5: Full image of Western Blotting analysis showing the time-dependent effects of PMA + IFNγ (inducer) on the expression of p-JNK. Figure S6: Full image of Western Blotting analysis showing the time-dependent effects of PMA + IFNγ (inducer) on the expression of t-JNK.

## Abbreviations

ANOVA	one-way analysis of variance
CGA	Chlorogenic acid
CQA	caffeoylquinic acids
DiCQA	Dicaffeoylquinic acids
ERK1/2	extracellular signal-regulated kinase 1 and 2
FBS	fetal bovine serum
JNK	c-Jun N-terminal kinases
IFNγ	interferon gamma
IL-6	interleukin-6
IL-8	interleukin-8
IBD	inflammatory bowel diseases
MAPK	mitogen-activated protein kinases
MEM	minimum essential medium
MTT	3-(4,5-dimethylthiazol-2-yl)-2,5-diphenyl tetrazolium bromide
NFκB	nuclear factor kappa-light-chain-enhancer of activated B cells
PMA	phorbol myristate acetate
P38	p38 isomers
TNF-α	tumor necrosis factor alpha
TEER	Transepithelial Electrical Resistance
