# Supplementary material for: Chlorogenic Acid (CGA) Isomers Alleviate Interleukin 8 (IL-8) Production in Caco-2 Cells by Decreasing Phosphorylation of p38 and Increasing Cell Integrity
Source: Int J Mol Sci. 2018 Dec 4;19(12):3873. doi: 10.3390/ijms19123873 (PMC6320834; doi:10.3390/ijms19123873)
Supplement: Supplementary file 1 [file ijms-19-03873-s001.docx]

**Supplementary Data for Liang and Kitts, 2018.**

**
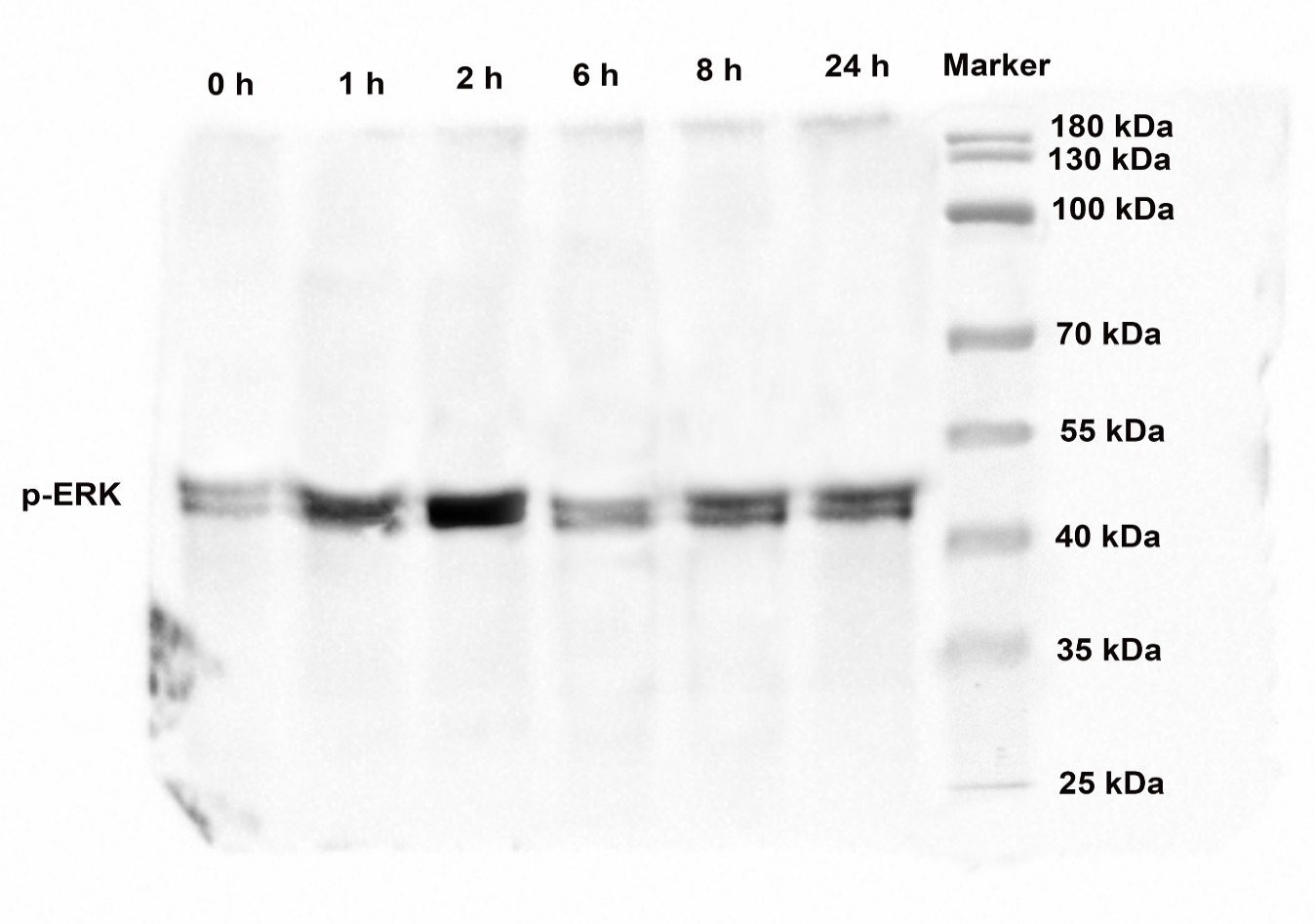
**

**Figure S.**1 Western blot to exam the time-dependent effects of PMA+IFNγ on the expression of p-ERK1/2 in Caco-2 cell. Caco-2 cells were collected after PMA+IFNγ challenge at 0 (lane 1), 1(lane 2), 2 (lane 3), 6 (lane 4), 8 (lane 5) and 24 (lane 6) hours.


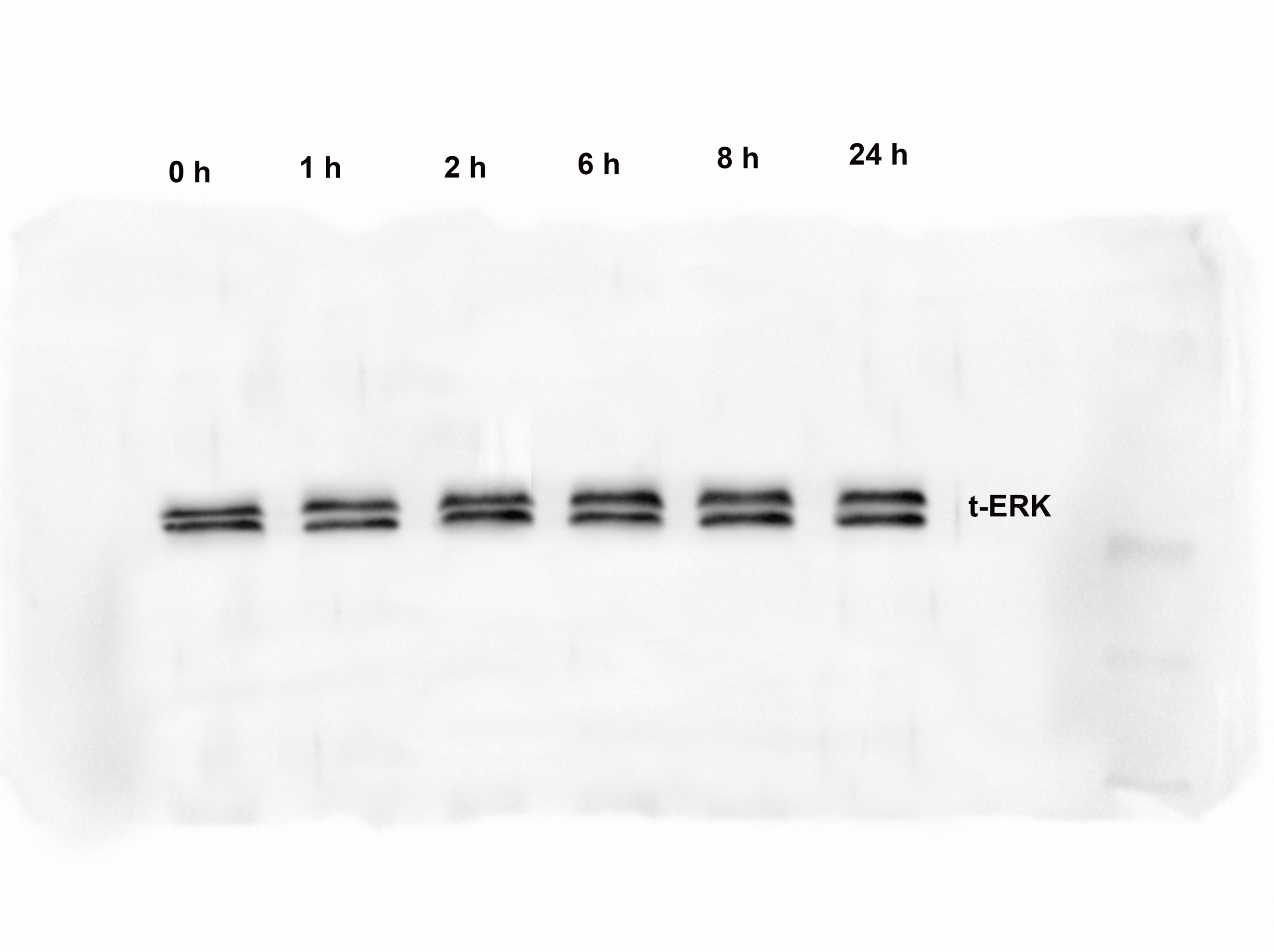


Figure S.2 Western blot to exam the time-dependent effects of PMA+IFNγ on expression of t-ERK1/2 in Caco-2 cell. Caco-2 cells were collected after PMA+IFNγ challenge at 0 (lane 1), 1(lane 2), 2 (lane 3), 6 (lane 4), 8 (lane 5) and 24 (lane 6) hours.


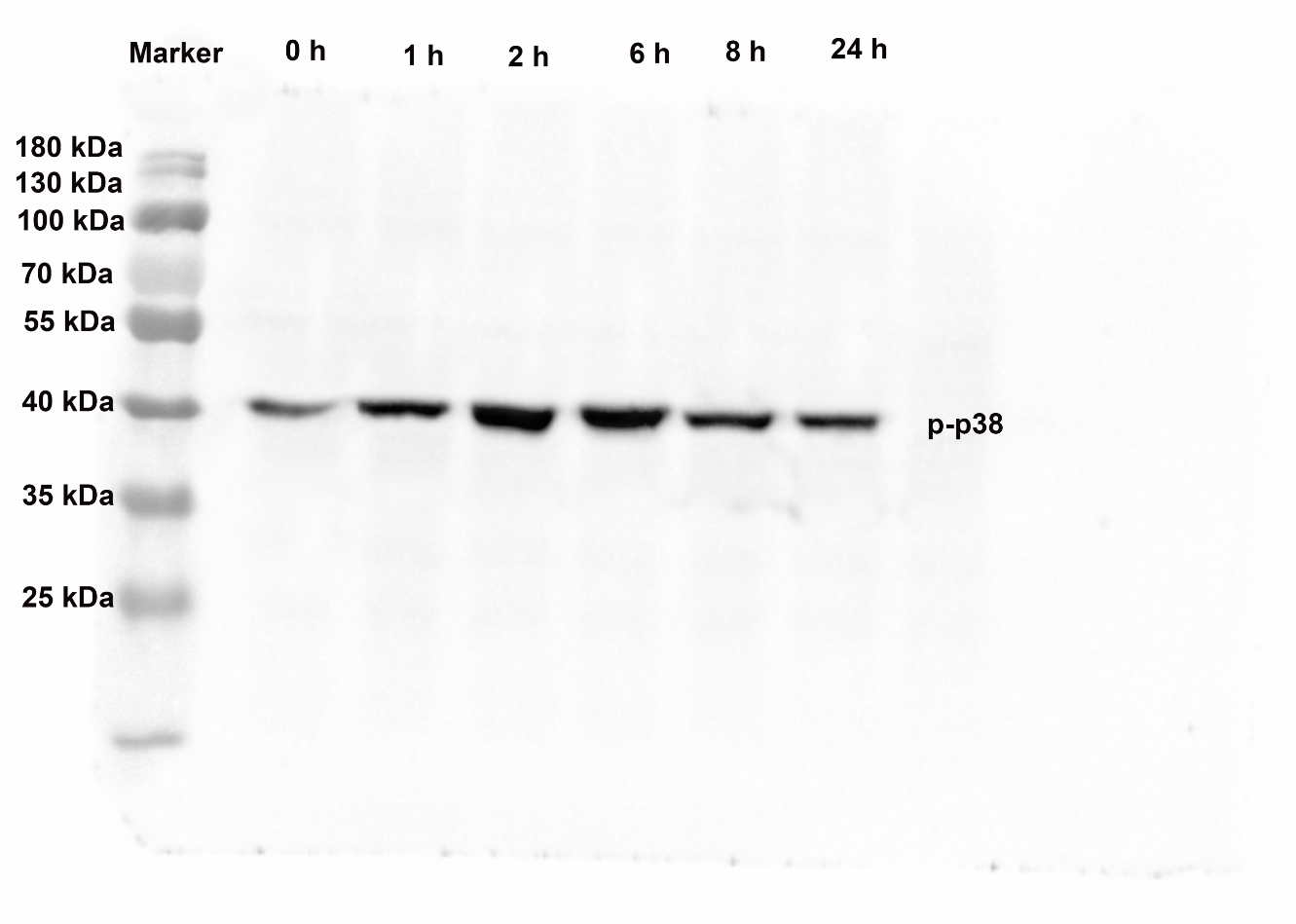


Figure S.3 Western blot to exam the time-dependent effects of PMA+IFNγ on the expression of p38 in Caco-2 cell. Caco-2 cells were collected after PMA+IFNγ challenge at 0 (lane 2), 1(lane 3), 2 (lane 4), 6 (lane 5), 8 (lane 6) and 24 (lane 7) hours. Lane 1 is the protein ladder marker.


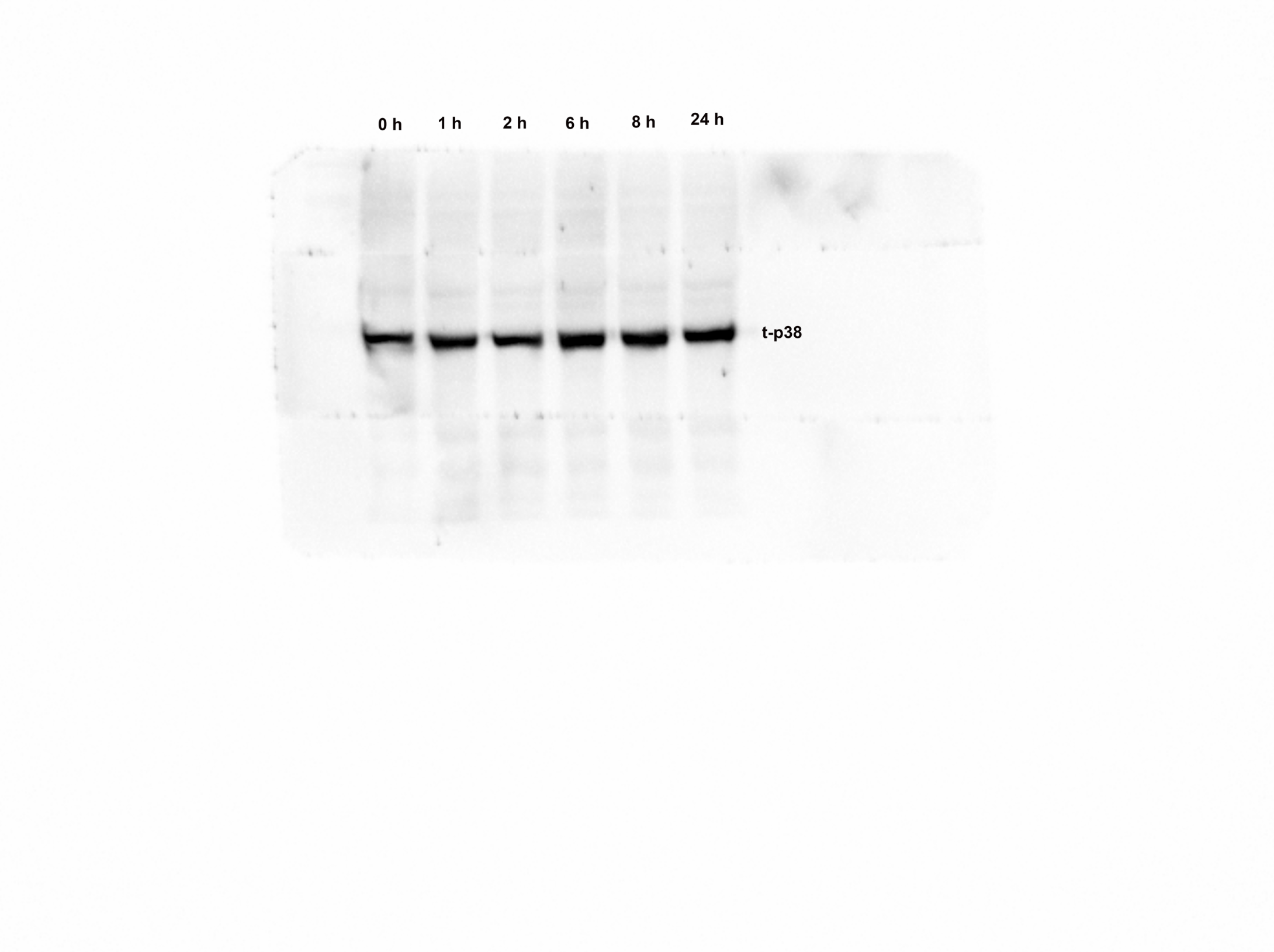


**Figure S.**4 Western blot to exam the time-dependent effects of PMA+IFNγ on expression of t-p38 in Caco-2 cell. Caco-2 cells were collected after PMA+IFNγ challenge at 0 (lane 1), 1(lane 2), 2 (lane 3), 6 (lane 4), 8 (lane 5) and 24 (lane 6) hours.


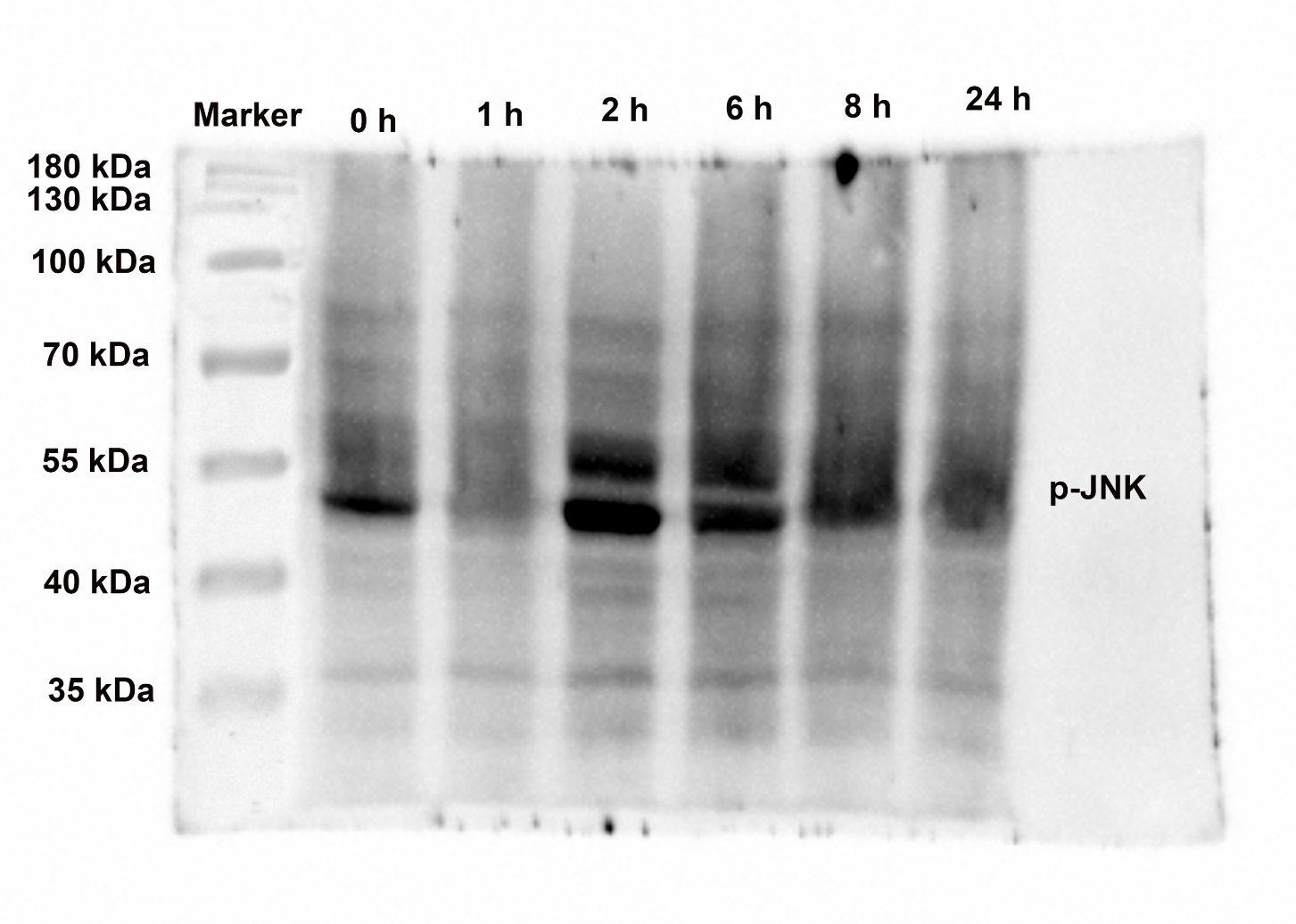


Figure S.5 Western blot to exam the time-dependent effects of PMA+IFNγ on the expression of p-JNK in Caco-2 cell. Caco-2 cells were collected after PMA+IFNγ challenge at 0 (lane 2), 1(lane 3), 2 (lane 4), 6 (lane 5), 8 (lane 6) and 24 (lane 7) hours. Lane 1 is the protein ladder marker.


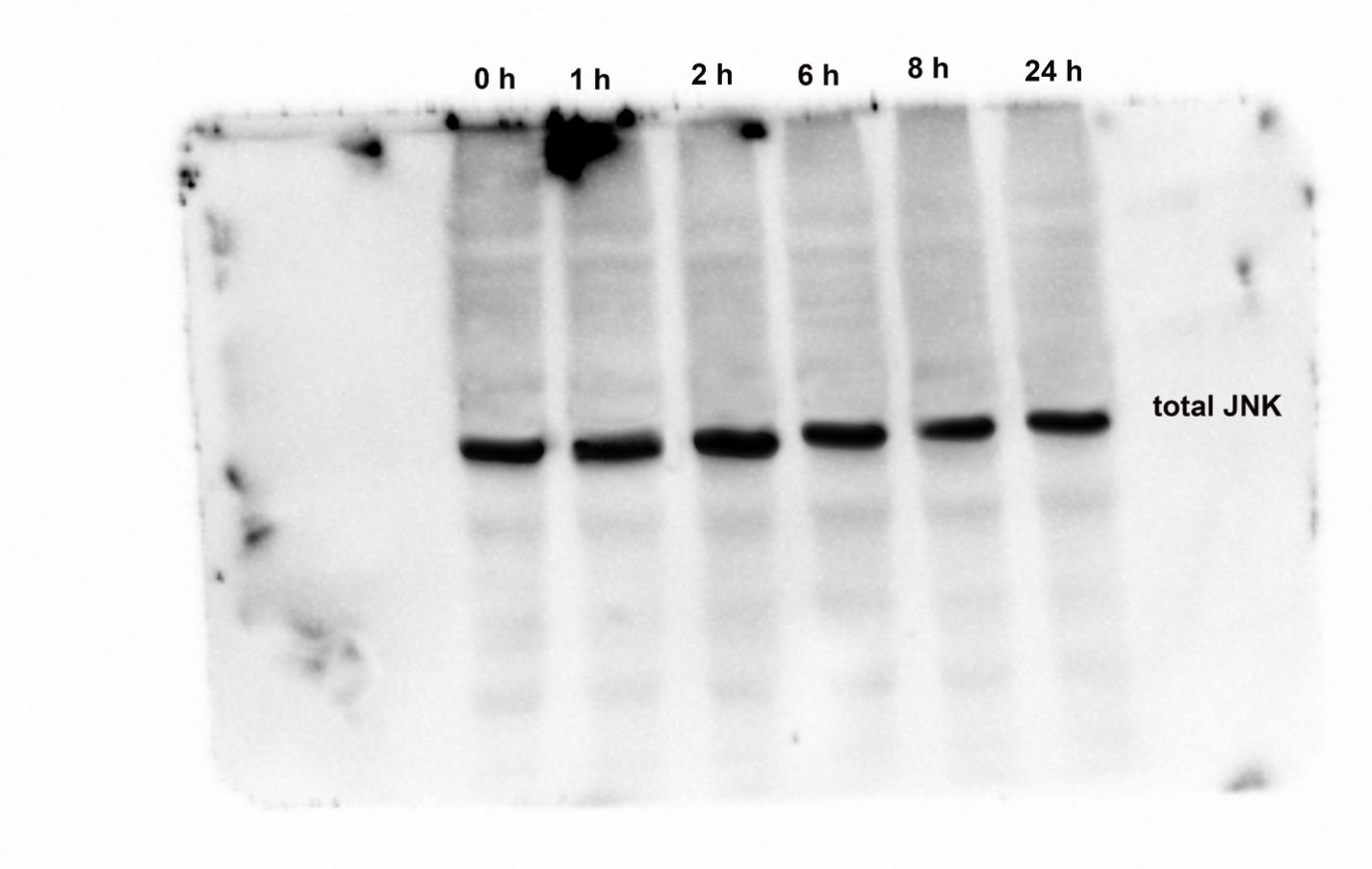


Figure S.6 Western blot to exam the time-dependent effects of PMA+IFNγ on expression of t-JNK in Caco-2 cell. Caco-2 cells were collected after PMA+IFNγ challenge at 0 (lane 1), 1(lane 2), 2 (lane 3), 6 (lane 4), 8 (lane 5) and 24 (lane 6) hours.
